# Predicting Drosha and Dicer Cleavage Sites with DeepMirCut

**DOI:** 10.3389/fmolb.2021.799056

**Published:** 2022-01-24

**Authors:** Jimmy Bell, David A. Hendrix

**Affiliations:** ^1^ School of Electrical Engineering and Computer Science, Oregon State University, Corvallis, OR, United States; ^2^ Department of Biochemistry and Biophysics, Oregon State University, Corvallis, OR, United States

**Keywords:** microRNA, microRNA biogenesis, machine learning, deep learning, genomics, long short-term memory network

## Abstract

MicroRNAs are a class of small RNAs involved in post-transcriptional gene silencing with roles in disease and development. Many computational tools have been developed to identify novel microRNAs. However, there have been no attempts to predict cleavage sites for Drosha from primary sequence, or to identify cleavage sites using deep neural networks. Here, we present DeepMirCut, a recurrent neural network-based software that predicts both Dicer and Drosha cleavage sites. We built a microRNA primary sequence database including flanking genomic sequences for 34,713 microRNA annotations. We compare models trained on sequence data, sequence and secondary structure data, as well as input data with annotated structures. Our best model is able to predict cuts within closer average proximity than results reported for other methods. We show that a guanine nucleotide before and a uracil nucleotide after Dicer cleavage sites on the 3′ arm of the microRNA precursor had a positive effect on predictions while the opposite order (U before, G after) had a negative effect. Our analysis was also able to predict several positions where bulges had either positive or negative effects on the score. We expect that our approach and the data we have curated will enable several future studies.

## Introduction

MicroRNAs (miRs) are a conserved class of endogenous small RNAs around 22 nucleotides (nt) in length. Mature microRNAs modulate a variety of different processes through post-transcriptional gene silencing, which results in either transcript degradation or translational inhibition (Liu, 2008). MicroRNAs are involved in a wide range of functions including cancer (both tumor-suppressor and oncogenic) (Zhang et al., 2007), development (Carrington and Ambros, 2003), stress response (Leung and Sharp, 2010), aging (Smith-Vikos and Slack, 2012), and circadian rhythms (Na et al., 2009). Nucleotide positions 2 through 8 on the mature microRNA, called the seed sequence, help direct the sequence-specific activity of the RNA-induced silencing complex (RISC), where it binds to a complementary strand on the 3′ UTR of an mRNA transcript. In some cases, microRNA may bind to target sites along CDS of RNA (Schnall-Levin et al., 2010; Zhang et al., 2018). Several CDS target-sites are known to suppress MicroRNA regulatory activity by acting as microRNA sponges (Ebert et al., 2007), including circular RNAs (Hansen et al., 2013), and long noncoding RNAs (Cheng and Lin, 2013). Other target-sites such as those found on a lncRNA called Cyrano can lead to target-directed miRNA degradation (TDMD) (Kleaveland et al., 2018; Han et al., 2020; Shi et al., 2020). ZSWIM8 ubiquitin ligase plays a role in TDMD by polyubiquinating Argonaut, which results in its proteolysis, thereby exposing the miRNA to degradation (Han et al., 2020; Shi et al., 2020).

**FIGURE 1:**
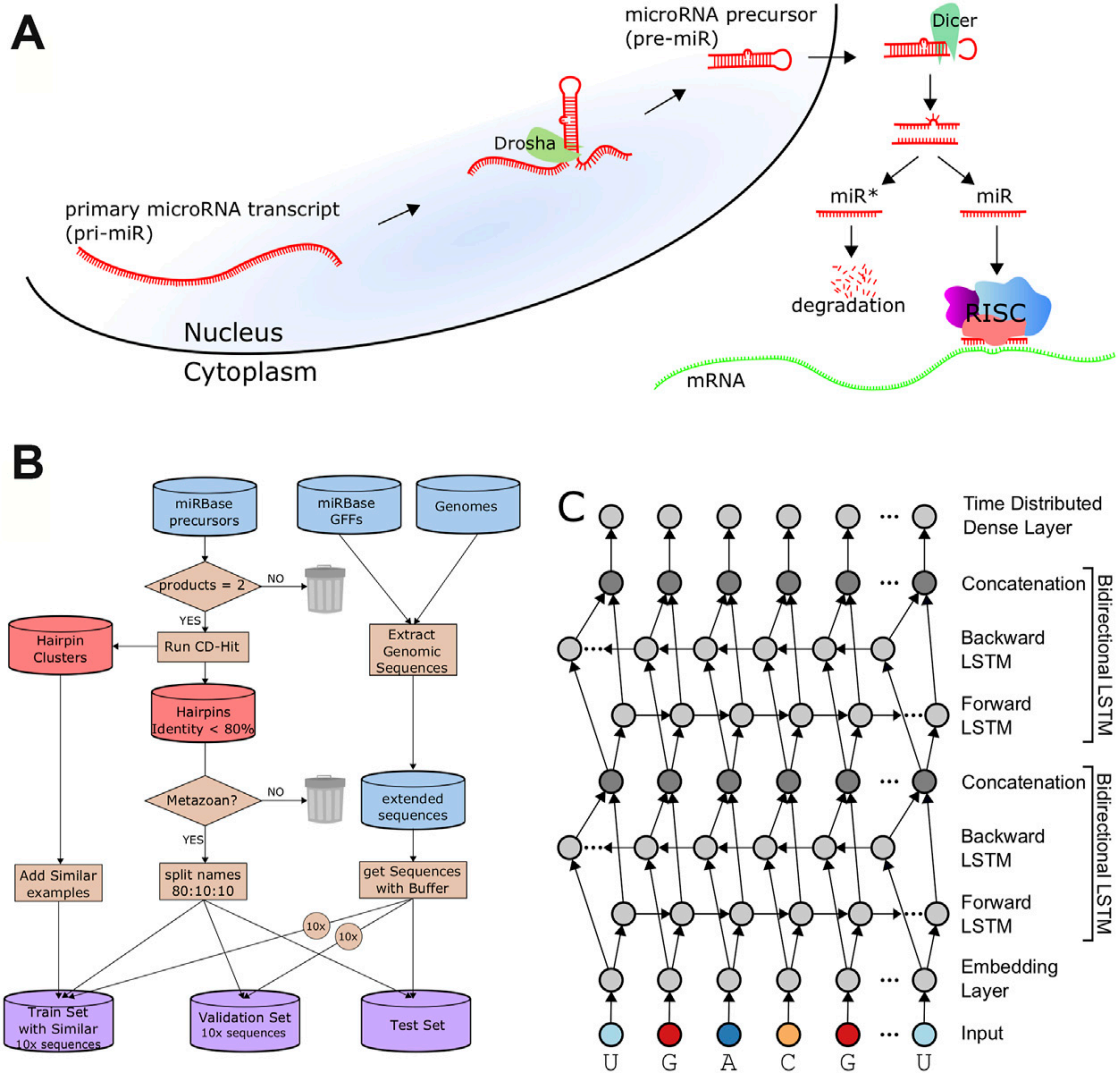
Data Processing and Architecture. **(A)**. A schematic of microRNA biogenesis showing that two cleavages of double-stranded RNA results in four cut sites to produce two mature products. **(B)**. Flowchart describing the generation of the train, validation, and test sets. **(C)**. The architecture of DeepMirCut consists of two bidirectional LSTMs, and a time-distributed dense layer.

The biogenesis of mature microRNAs (Figure 1A) begins with the transcription of a primary miRNA (pri-miRNA) transcript by RNA Polymerase II (Lee et al., 2004; Zhou et al., 2007), or in rare cases RNA Polymerase III (Borchert et al., 2006). The microprocessor complex associates with the hairpin, whereby the action of the component enzyme Drosha produces a double-stranded cleavage that results in the microRNA precursor (pre-miR), leaving a 2-nt overhang on the 3′ end (Lee et al., 2003; Gregory et al., 2004). Exportin-5 associates with the 3′ overhang and transports the precursor from the nucleus to the cytoplasm (Yi et al., 2003). In the cytoplasm, Dicer removes the hairpin loop through an additional double-stranded cleavage. Taken together, the activity of these enzymes results in four distinct cleavage sites (here also called “cut sites”) of the pri-miRNA transcript and produce a double-stranded duplex consisting of a 5 and 3′ mature product (Figure 1A). Dicer passes the duplex to Argonaut, a core enzyme of RISC, which binds to only one of the strands while the other is typically degraded.

Several tools have been developed for the analysis of microRNAs, but these approaches are limited by two challenges. First, these methods focus on microRNA discovery, but very little has been done to predict the locations of the cut sites resulting from microRNA biogenesis, especially in the absence of deep sequencing data. Second, these microRNA discovery tools, such as miRWoods (Bell et al., 2019), miRTRAP (Hendrix et al., 2010), miReNA (Mathelier and Carbone, 2010), miRDeep (Friedländer et al., 2008), miRDeep2 (Friedländer et al., 2012), miReap (Chen et al., 2009), and miRAnalyzer (Hackenberg et al., 2009), use score-based or machine learning approaches to classify loci as microRNAs, and therefore rely heavily on feature engineering. Although the tools benefit from features that are easily interpretable, feature engineering can be laborious.

Deep learning approaches overcome the need for feature engineering by learning the features from more basic input data. Several deep learning approaches such as convolutional neural networks (CNNs) (Do et al., 2018) and recurrent neural networks (RNNs) (Park et al., 2016; Cao et al., 2018) have been used for microRNA classification. While these approaches have addressed the limitations of feature engineering, they only predict loci and do not perform cleavage-site prediction. RNNs, such as Long Short-Term Memory (LSTM) networks, have been used in natural language processing applications such as named-entity recognition (Lample et al., 2016) and part-of-speech tagging (Wang et al., 2015), which are similar tasks to cleavage site recognition. Motivated by the challenges of microRNA analysis and the success of deep learning applications for NLP, we created DeepMirCut, an LSTM-based algorithm that predicts Dicer and Drosha cleavage sites within microRNAs. DeepMirCut predicts the locations of the four cut sites of Drosha and Dicer from an input RNA sequence. Moreover, because most microRNA annotations stop at Drosha cleavage sites and do not include the larger flanking genomic sequence, we curated a new enhanced microRNA sequence data set that includes 300-base-pair (bp) flanking sequence.

While most microRNA tools focus on homologous and novel microRNA discovery, few tools have been developed to predict cleavage sites involved in microRNA biogenesis, and no tools have been developed to predict Drosha sites from primary transcript sequences. Some tools have been developed to address the similar task of Dicer cut sites from shorter sequences. PHDCleav is an support vector machine (SVM) designed to identify Dicer cut sites on human microRNA precursors (Ahmed et al., 2013). While PHDCleav performs well on a test set, when the SVM is applied in a sliding window across the entire precursor, the cut site predictions are on average 3.1 nucleotides offset from the annotation (Ahmed et al., 2013). LBSizeCleav is similar but adds features describing the length of loop and bulge structures (Bao et al., 2016). LBSizeCleav performs with greater accuracy than PHDCleav at finding cleavage sites within 1nt of the annotated site, but has lower accuracy when more of an offset is allowed (Bao et al., 2016).

## Results

### Dataset Generation

For our analysis, we processed microRNA annotations from miRBase with the corresponding genomic sequences to extract microRNA precursor sequences as well as up to 300-nt flanking genomic sequence. We extracted flanking sequences shorter than 300 nt in cases where they overlapped neighboring microRNA or there was not enough genomic sequence surrounding the annotation. We refer to the precursor and flanking sequence as an “extended sequence”. Our data processing resulted in a collection of 34,713 extended sequences for both metazoan and plant species.

Because plant and animal (metazoan) microRNA biogenesis is very different (Kurihara and Watanabe, 2004; Axtell et al., 2011), and because more data is available for metazoa to train deep learning models, we focused this current study on precursors from metazoan species having both mature microRNAs (5 and 3′) annotated in miRBase, which consists of 11,296 records. Precursor sequences with an identity threshold of at least 80% to other sequences were excluded from the set using CD-Hit (Fu et al., 2012) in order to ensure low similarity between the training, validation, and testing sets. An 80:10:10 split was used to produce a training set with 3,923 examples, validation set with 490 examples, and test set with 491 examples. To increase our training examples, we added back sequences that CD-Hit had identified as similar to those in the training set but were below the sequence identity threshold of the validation and testing sets, which increased the training set to 8,491 examples. We compared each sequence in the training set with sequences in the validation and testing sets to verify that an identity of less than 0.8 was maintained for sequences between sets as demonstrated in Supplementary Figure S1. Random lengths of flanking genomic sequence between 30 nt and 50 nt were included with each of the precursors for the training, validation, and testing sets. An augmented training set with 84,910 examples and an augmented validation set with 4,900 examples were generated by randomly selecting 9 additional random flanking genomic sequence lengths for each precursor (Figure 1B).

### Model Architecture

We trained three different sets of models defined by the type of input data. First, model 1 was trained on only the extended RNA sequence. Second, model 2 was trained using the RNA sequence and secondary structure dot-bracket sequence. RNAfold (Lorenz et al., 2011) was used to predict the secondary structure of the entire extended RNA sequence, to provide the dot-bracket (Hofacker et al., 1994) sequence for each RNA within each of the train, test, and validation sets. Finally, for model 3, we further annotated the sequence using its bpRNA structure array (Danaee et al., 2018) to provide a single-character code for each position, such as whether the nucleotide was on a bulge, internal loop, or hairpin loop. The DeepMirCut software combines base-pairs identified by RNAfold with loop-type identified by bpRNA into a single modified bpRNA sequence using “L” and “R” to refer to 5′ (left) and 3′ (right) nucleotides participating in base pairs. Model 3 was trained using the RNA sequence and this enhanced bpRNA structure array sequence.

**FIGURE 2:**
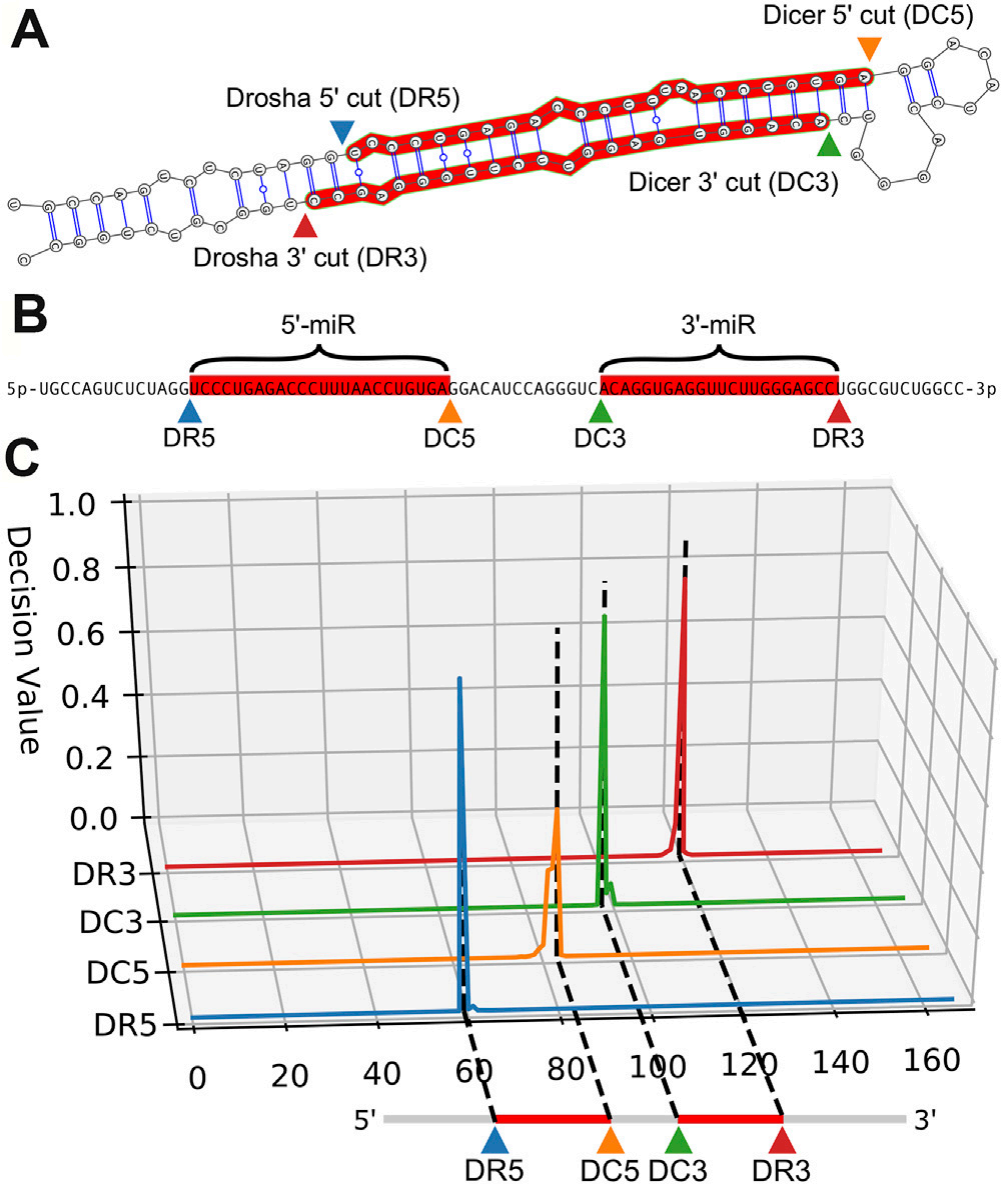
Example cleavage site identification for hsa-mir-125a. **(A)**. Dicer and Drosha cut sites relative to the secondary folding structure of hsa-mir-125a. The enzymes Dicer and Drosha cleave the hairpin resulting in the two mature microRNA sequences highlighted in red. **(B)**. A linear representation of the same RNA sequence, with the mature products highlighted in red, and labels indicating cut sites and mature products. **(C)**. Quantitative curves show the predictions of DeepMirCut along the length of the extended precursor sequence for the Drosha and Dicer 5′ and 3′ labels.

The architecture includes an embedding dimension, a dropout layer, two bidirectional LSTM layers, and a time-distributed layer, which is a dense layer that provides outputs for each position of the input sequence. The time-distributed layer outputs a set of 5 values for each nucleotide which represent weights for a Drosha cut on the 5′ arm (DR5), a Drosha cut on the 3′ arm (DR3), a Dicer cut on the 5′ arm (DC5), a Dicer cut on the 3′ arm (DC3), or no cut site present (O). By default, DeepMirCut labels the position with the maximum weight for DR3, DR5, DC3, and DC5 as a cleavage site, but the O-sites are not labeled (Figure 1C). Labeling is done in this way so that each cut site will only be labeled once, rather than labeling using that maximum weight at each position, which could result in cut sites being labeled more than once or not at all. See Figure 2 for an example of DeepMirCut predicting cleavage sites for hsa-mir125a.

### Evaluation Metrics and Tuning

Precision, recall, and F-score are often used to evaluate the performance of machine learning models applied to binary classification tasks. However, when evaluating DeepMirCut each of these measurements ends up being the same since a single label is predicted for each cleavage site. For this reason, we have opted to use perfect match fraction (PMF) and position shift error (PSE) to measure performance (see Performance Metrics in Methods.)

Hyperparameters were tuned to identify the best parameter combinations for models trained using each of three input options for DeepMirCut. The top 10 architectures identified through tuning were each evaluated with 20 replicates to identify parameters resulting in the best median PMF (Supplementary Figures S2–S4). All models were evaluated using the augmented validation set. The parameter combinations that showed the best performance during tuning are shown in Table 1.

**TABLE 1:** Tuned parameters for each DeepMirCut model and type of input.

Input Type	Nucleotide sequence only	Nucleotide and dot-bracket sequence	Nucleotide and bpRNA sequence
Embedding layer	96 units	32 units	32 units
Dropout	0.315	0.213	0.417
Bi-LSTM layer 1	320 units	64 units	128 units
Bi-LSTM layer 2	192 units	256 units	320 units
Learning rate	3.2 10^–3^	1.91 10^–3^	3.57 10^–3^
Epsilon (10^x^)	−7.56	−6.79	−6.87

**FIGURE 3:**
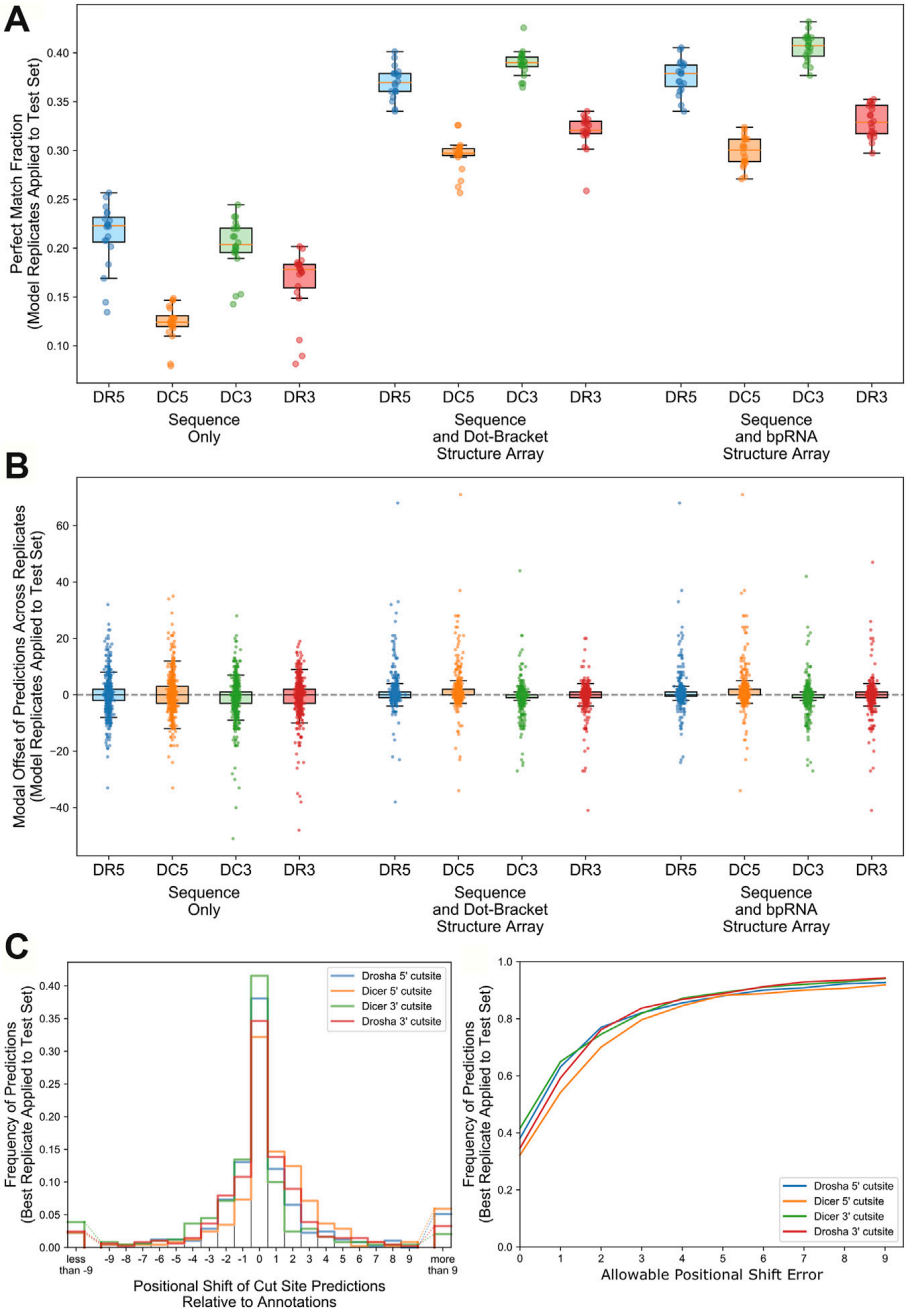
Comparison of performance for replicates trained with the best parameter combinations for each input type. **(A)**. Boxplot comparing the perfect match fraction found when each model replicate was run against the test set. **(B)**. Boxplot showing the modal offset of each prediction across all model replicates. **(C)**. Histogram showing the frequency of positional shifts of predicted cut sites relative to their annotated locations for the best replicate trained using nucleotide, RNAfold, and bpRNA structure array. **D.** A line plot showing the fraction of cut sites identified within varying distances from the annotations for the best replicate trained on nucleotide, RNAfold, and bpRNA structure array.

Models trained on RNA sequence only, RNA sequence and dot-bracket sequence, and RNA sequence and bpRNA structure array were evaluated against the test set using the optimum parameter combinations for each type of input. Replicates that were trained with the sequence and bpRNA structure array resulted in the highest median PMF for each cleavage site (Table 2 and Figure 3A) and the lowest position shift error for dicer cleavage sites (Table 2 and Supplementary Figure S5A). A boxplot showing the distributions of the modal offset between the cleavage site predicted by each replicate and the annotated cut sites for each example in the test set is shown in Figure 3B.

**TABLE 2:** Median, best replicate, and ensemble performance metrics for each type of cut site and input. The best performance for each column is indicate in bold, i.e. highest PMF or lowest PSE.

Model	DR5		DC5		DC3		DR3	
	PMF	PSE	PMF	PSE	PMF	PSE	PMF	PSE
Nucleotide sequence only (median)	0.223	4.998	0.124	5.147	0.204	4.85	0.178	4.776
Nucleotide and dot-bracket (median)	0.37	2.657	0.297	2.962	0.39	2.385	0.321	2.385
Nucleotide, and bpRNA (median)	0.379	2.687	0.3	2.929	0.407	2.295	0.329	2.425
Sequence and bpRNA (best replicate)	0.381	2.658	0.322	3.055	0.415	2.165	0.346	2.436
Sequence and bpRNA (ensemble)	**0.45**	**2.426**	**0.354**	**2.819**	**0.47**	**1.994**	**0.389**	**2.037**

### Best Performing Replicate

We identified the best-performing replicate trained on nucleotide and enhanced bpRNA structure array based on average PMF for all cut sites when evaluated against the validation set. Hereafter, we refer to this best model as “DeepMirCut”. We tested the performance of DeepMirCut on the test set and found that it performed best when identifying the DR5 and DC3 cut sites, which are the cut sites that release 5′-end of the mature microRNA sequences during microRNA biogenesis. The PMF for the DR5 and DC3 cut sites were 0.381 and 0.415 respectively, and the PSE for the DR5 and DC3 cut sites were 2.658 and 2.165 respectively (Table 2). Predictions for the DR5 and DC3 cut sites also had higher decision values than other cut sites showing that the algorithm predicted these cuts with greater confidence (Supplementary Figure S5B). Most predictions from DeepMirCut fell within one nucleotide of the annotated cleavage sites. (Figures 3C,D). When applied to the test set, this model also performed better than the median PMF for all replicates (Table 2).

### Point Mutation Analysis

**FIGURE 4:**
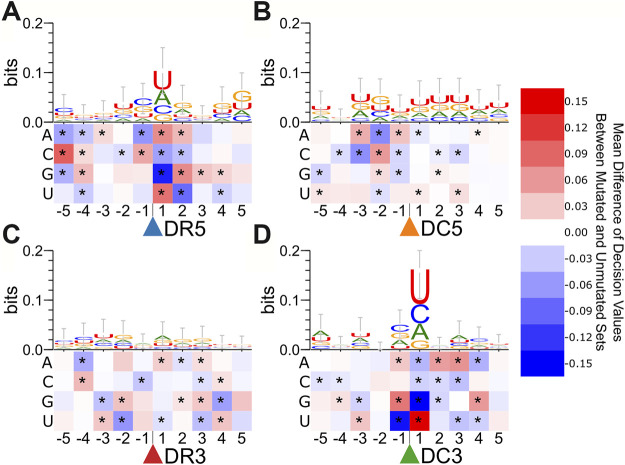
Point mutation analysis for nucleotides surrounding cut sites in the test set. Heatmaps show the average change in decision value due to point mutations for nucleotides surrounding cleavage sites of **(A)**. Drosha on the 5′ arm, **(B)**. Dicer on the 5′ arm, **(C)**. Drosha on the 3′ arm, and **(D)**. Dicer on the 3′ arm.

We performed a point-mutation analysis on the nucleotides surrounding each cut site to interpret the sequence features learned by DeepMirCut (Figure 4 and Supplementary Figure S6). The effect on scores for the Dicer cut site on the 3′ arm of the precursors was the most pronounced. A guanine nucleotide before and a uracil nucleotide after Dicer cleavage sites on the 3′ arm had a positive effect on predictions while the opposite order (U before, G after) had a negative effect. Uracil had the highest information content 1 nt downstream from the cleavage site, as indicated by the sequence logo (Figure 4B), and previous studies (Hu et al., 2009).

**FIGURE 5:**
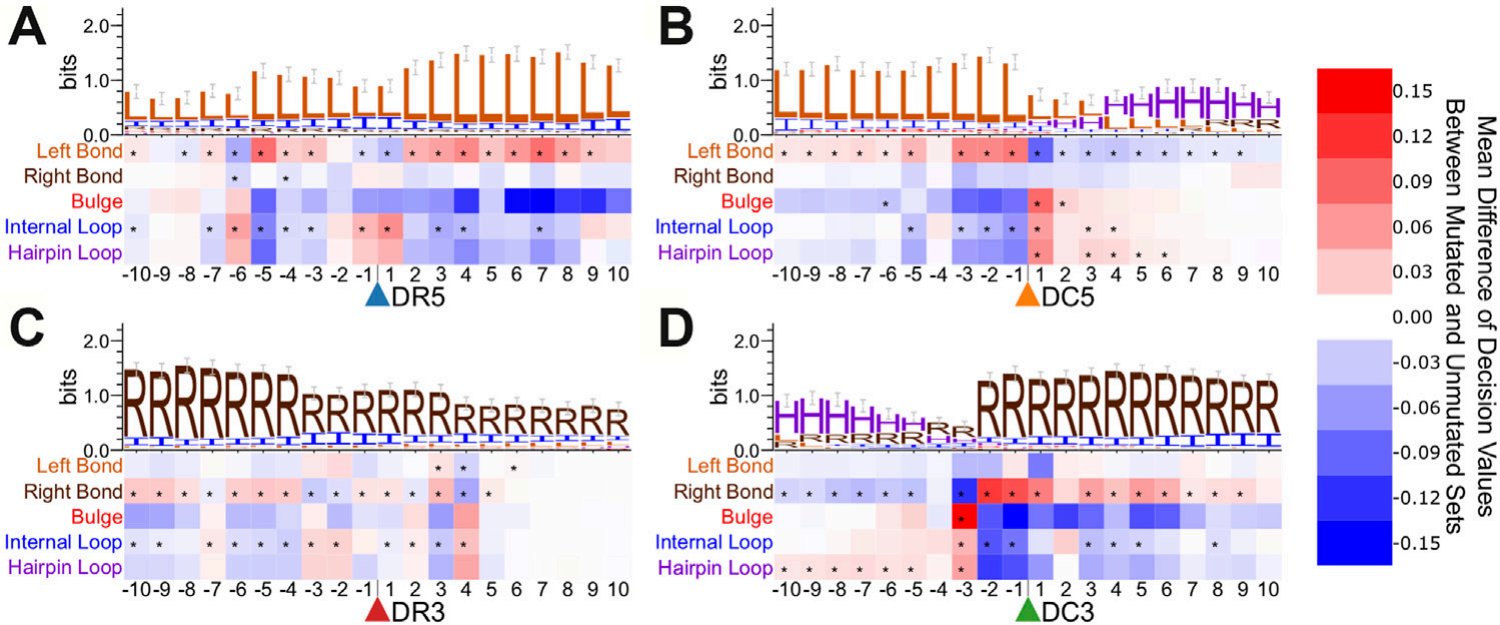
Point mutation analysis for bpRNA sequence surrounding cut sites in the test set. Heatmaps show the average change in decision value due to point mutations within the enhanced bpRNA sequence surrounding cleavage sites of **(A)**. Drosha on the 5′ arm, **(B)**. Dicer on the 5′ arm, **(C)**. Drosha on the 3′ arm, and **(D)**. Dicer on the 3′ arm.

We also performed a secondary structure point mutation analysis for the enhanced bpRNA structure array sequence (Figure 5 and Supplementary Figure S7). Asterisks indicate statistically significant score changes using a paired difference t-test with a Bonferroni multiple test correction. A bulge occurring 3 nt upstream had a positive influence on the identification of Dicer cleavage sites on the 3′ arm (Figure 5D). On the 5′ arm prediction performance improved when a bulge was present 1 nt downstream, but not 1-2nt upstream from the Dicer cleavage site (Figure 5B).

We further performed the same type of point mutation analyses on a specific conserved family. We examined members of the let-7 family, and observed many consistent trends when compared with the metazoan microRNAs as a whole (Supplementary Figures S8, S9). For example, we observe a strong uracil bias for the position immediately after the 5′ Drosha cut site and a preference for uracil surrounding the DC5 cut sites. Notable differences for let-7 include a preference for C after the DC3 cut site and greater sequence conservation around the DR3 cut site than is observed in the test set. Consistent with the general structural trends (Figure 5), we observe a strong preference for a bulge at position -3 relative to the DC3 site. The structure point mutations for let-7 show a stronger preference for a bulge immediately 3′ of the DR5 cut site and several positions that strongly favor internal loops, which may point to family-specific structural preferences.

### Ensemble Approach

**FIGURE 6:**
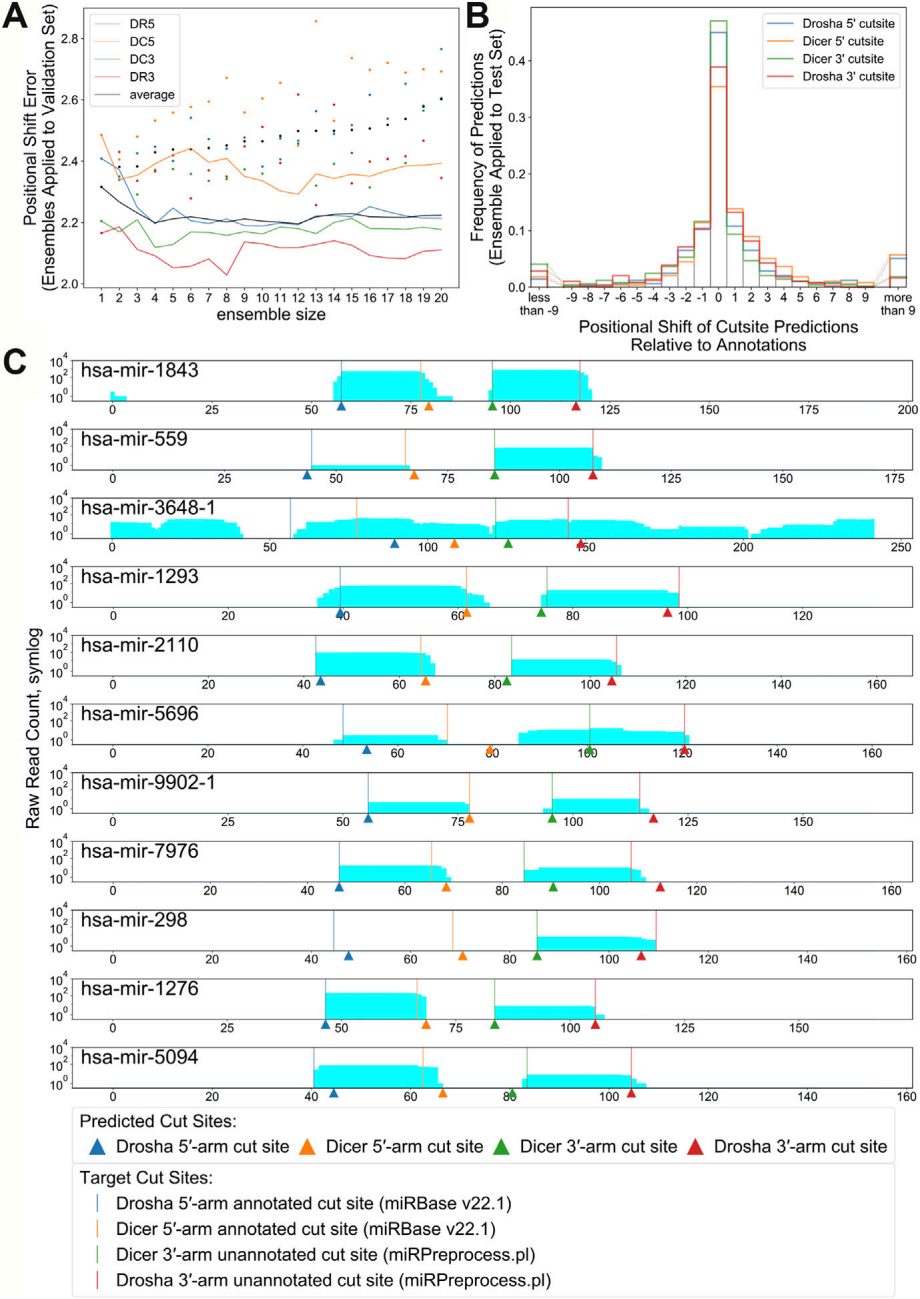
**(A)**. Positional Shift Error found when ensemble bpRNA is applied to the validation set for ensembles of increasing size (dots represent PSE for individual replicates being added to the ensemble). **(B)**. Frequency of predictions relative to each cut site when ensemble bpRNA is applied to the test set with an ensemble size of 12. **(C)**. Mapped read counts and cut site predictions for known microRNA precursors with unannotated microRNAs on the 3′ arm. Vertical lines show the position of cut sites that are either annotated in miRBase or predicted using the miRPreprocess.pl script from miRWoods. Arrows show the location of cut sites predicted by ensemble DeepMirCut.

The top 12 best performing replicates trained on RNA sequence and enhanced bpRNA structure array were combined into an ensemble. The number of replicates was chosen so that it resulted in the highest PMF and lowest PSE when tested against the validation set (Figure 6A and Supplementary Figure S5C). Hereafter, we refer to this model as “ensemble DeepMirCut.” Ensemble DeepMirCut was applied to the test set and performed better than the single model version of DeepMirCut for each cleavage site. Ensemble DeepMirCut performed best on the DC3 cut site with a PMF of 0.47 compared to a PMF of 0.415 for the single model version of DeepMirCut. Like the single model version, Ensemble DeepMirCut had better performance when identifying cut sites that corresponded to the 5′-end of mature microRNAs (Table 2). Most predictions fell within 1 nt of the annotated cleavage sites (Figure 6B). Ensemble DeepMirCut can identify the Dicer cut site on the 3′ arm with an average PSE of 1.994 over our test set of metazoan miRs (Table 2).

### Testing Against Mirtrons

Mirtrons are Drosha-independent microRNAs that use intron splicing for removal from primary transcripts, and would not fit in our intended application of DeepMirCut. Mirtron labels are not available in miRBase, and databases that exist do not cover all species in our data set; therefore, we included mirtrons in our test and training sets. We used mirtronDB to identify mirtrons for annotated species in our dataset (Da Fonseca et al., 2019). Our training set was composed of 4.66% mirtrons, and our test set consisted of 9.6% mirtrons from mirtronDB. We tested whether mirtrons show an increase in DeepMirCut prediction error by splitting our test set between mirtrons and canonical microRNAs. When comparing Drosha cleavage site prediction, we found that DeepMirCut predicted DR5 sites for mirtrons with a PSE of 3.56 and predicted DR5 sites for canonical microRNA with a PSE of 2.57. Similarly, ensemble DeepMirCut predicted cut sites for DR5 with a PSE of 2.86 for mirtrons, and 2.38 for canonical miRs (Supplementary Table S2). Unexpectedly, DR3 sites showed the lowest PSE when evaluated on mirtrons.

### Testing Against microRNAs with Questionable Validity

We further tested whether prediction error would increase for microRNAs with questionable status. To evaluate this, we downloaded a dataset of 177 questionable mouse microRNAs from Chiang *et al* (Chiang et al., 2010). MicroRNAs and buffer sequence were extracted from the NCBIM37 mouse genome assembly using the GFF from miRBase version 14. Fourteen microRNAs were excluded due to having a single product crossing into the hairpin loop, which made determining the side of the product ambiguous. DeepMirCut and ensemble DeepMirCut both scored with a PSE of more than 5 for each cut site (Supplementary Table S3). Due to the unusually high PSE, this observation corroborates that many of the microRNA in the dataset are not true miRs.

### Cleavage Site Prediction for Unannotated Mature microRNAs

Although DeepMirCut was trained on microRNA annotations with two mature microRNA products, we reasoned that we could predict the location of the missing product for precursors with only one annotated microRNA product. We collected precursor annotations from miRBase with only one annotated mature product, but that had mapped reads from small RNA deep sequencing data. Using the ensemble, we tested the performance of DeepMirCut on precursors with only one annotated microRNA by generating sets with the annotated microRNA either on the 3′ arm or the 5′ arm. Cleavage sites on the arm opposite to the annotated microRNAs were assumed based on read stacks from small RNA sequencing data (see Methods) and were used to assess performance. Ensembled DeepMirCut was able to predict cuts that corresponded to unannotated microRNA where small RNA sequencing reads had mapped. (PMF_DR5_ = 0.444, PMF_DC5_ = 0.444, PMF_DC3_ = 0.545, PMF_DR3_ = 0.364; PSE_DR5_ = 5.000, PSE_DC5_ = 3.778, PSE_DC3_ = 1.364, PSE_DR3_ = 1.818). (Figure 6C and Supplementary Figure S10).

### DeepMirCut Compared to Other Approaches

We compared DeepMirCut to PHDCleav and LBSizeCleav using the implementation from *Bao et al.* (https://sunflower.kuicr.kyoto-u.ac.jp/∼houu/LBSizeCleav/index.html) (Bao et al., 2016). We designed an experiment to perform as direct of a comparison of these approaches as possible, utilizing test conditions based on the original paper, which compares the true cut site to 6 nt downstream.

We trained and tested PHDCleav and LBSizeCleav on the metazoan training set described above, which removes sequence replicates since these would result in redundant sequence and structural patterns. DeepMirCut and ensemble DeepMirCut performed with a much higher specificity but did not outperform the accuracy or sensitivity of the best models for PHDCleav or LBSizeCleav (Supplementary Table S4). It is important to note that we had to change our output to compare with PHDCleav and LBSizeCleav. The authors of PHDCleav used a sliding window approach and they reported a PSE of 3.1 for their best model (Ahmed et al., 2013). In contrast, when detecting the DC3 cut site DeepMirCut performed with a PSE of 2.165 and Ensemble DeepMirCut with a PSE of 1.994 (Table 2). However, we did not have the code available to analyze the PSE of PHDCleav or LBSizeCleav further.

## Discussion

Few studies have predicted Drosha cut sites from sequence, but the importance of Drosha in microRNA biogenesis is best illustrated by experiments that show that knocking-out Drosha abolishes microRNA biogenesis, while knocking-out Dicer only reduces the abundance of mature microRNAs (Kim et al., 2016). We describe the training, testing, and evaluation of DeepMirCut for the site-labeling of Dicer and Drosha cleavage sites on extended precursor sequences that includes surrounding genomic sequences. Previous methods such as PHDCleav and LBSizeCleav address similar tasks, yet differ from the work presented here for several reasons. First, they do not predict Drosha cut sites. Second, they were only trained and tested on human sequences. Deep learning methods require much larger training sets; therefore, we worked with all available metazoan primary sequences. Third, these approaches are applied to microRNA precursor sequences, but DeepMirCut is applied to extended precursor sequences that incorporate the context from longer portions of the primary transcript in order to predict Drosha sites. Because these approaches address a different task and are applied to different input sequences, they cannot be directly compared. DeepMirCut predicts both Dicer and Drosha cleavage sites on full-length extended precursor sequences that include flanking sequence of randomly-sampled length. Our experiments with annotations from miRBase show that DeepMirCut labels cleavage sites with close average proximity when applied to full-length extended sequences, which is a more difficult task than previous classification approaches.

We expect that the improved performance comes from the multi-layered recurrent neural network, and the more-comprehensive input data, which includes both nucleotide sequence and annotated secondary structure sequences with dot-bracket and enhanced bpRNA structure array. Although secondary structure improves performance, we found that DeepMirCut can predict moderately-well based on sequence alone, suggesting it is not completely relying on structural information about the loop for its predictions. This is consistent with the fact that point-mutation analysis reveals strong changes in score due to perturbations to sequence alone. It is known that cleavage of microRNA precursors tends to include uracil residues and exclude guanine residues at the ends of mature microRNAs when cut by Dicer (Starega-Roslan et al., 2015a; Starega-Roslan et al., 2015b) and Drosha (Starega-Roslan et al., 2015b). Our analysis shows that point mutations to uracil at each end of both mature products had a positive effect on decision value. Point mutations resulting in a guanine at either end of the mature products except DR3 had a negative effect on decision value. Adenine residues are frequently found 2 nt upstream from the dicer cleavage site on the 5′ arm (Starega-Roslan et al., 2015b). In our point mutation analysis, adenine at this position had a positive effect on decision value. The addition of structural context to the training and testing set further improved the performance of DeepMirCut. It has been shown biochemically that the hairpin loop position (Gu et al., 2012) and the locations of bulges and other unpaired nucleotides (Feng et al., 2012) may help direct the function of Dicer. Our point-mutation analysis of regions beyond the precursor corroborates previous genome-wide experimental studies of Drosha cleavage sites (Kim et al., 2017). However, our structural heatmap allows us to visualize the importance of certain loop types (internal loops vs bulges) beyond just paired vs unpaired. We note two limitations in our structural point-mutation analysis (Figures 4, 5). First, the analysis is not necessarily interpretable for double-mutations, as only the results of single mutations were measured. For example, while single-nucleotide internal loops are favorable when adjacent to Drosha 5′ cut sites, we expect it is not favorable to have a two-nucleotide internal loop spanning the cut site. Second, these point-mutation heat maps are limited to structure and sequence data available in the training set.

Central to microRNA function is the seed sequence, which is necessary for the RISC to target specific mRNAs and is defined relative to the 5′ end of the mature microRNA. Consistent with these functional requirements, we observed that DeepMirCut performed better at the identification of cleavage sites corresponding to the 5′ ends of mature microRNAs compared to their 3′ ends. These data support the idea that Dicer and Drosha are directed to these cleavage sites by sequence and structural information. A possible reason for this is a greater variability of cut sites at the 3′ end of microRNAs (Neilsen et al., 2012), which makes training and testing is more difficult for these sites. Although this is true separately for the 5 and 3′ arms (DR5 is better than DC5 and DC3 is better than DR3), we also note that DR3 is the second most accurately predicted cut site and does not correspond to the 5′ end of a mature microRNA. For reasons that are unclear, DR3 sites were also accurately predicted for mirtrons. DeepMirCut was able to predict DR3 sites accurately for both canonical miRs and mirtrons despite the fact that we do not observe obvious trends in our logos (Figures 4, 5) and that Drosha is not involved in mirtron biogenesis.

As we noted previously (Bell et al., 2019), structure prediction of microRNA precursors is sensitive to the length of the sequence, and adding the 30- to 50-nt flanking sequence may add variability to structure prediction. The possibility remains that improved secondary structure prediction may improve performance of DeepMirCut.

While DeepMirCut is a valuable tool for the automatic prediction of Drosha and Dicer sites, it is not a substitute for experimental methods, which provide empirical evidence of cut site locations, and can directly detect isomirs (Neilsen et al., 2012; Starega-Roslan et al., 2015a). That said, DeepMirCut is trained on datasets built from multiple experimental data sources, and may uncover common principles describing the most frequently occurring cut sites in microRNA biogenesis. For this reason, DeepMirCut can be used in the design of synthetic miRs so as to match typical sequence and structural features observed in endogenous microRNAs, or to assist in the prediction of novel microRNAs in metazoan genomes that lack a microRNA annotation. Furthermore, we have shown that DeepMirCut predictions for microRNAs of questionable validity show a substantially higher PSE than more confident annotations, and therefore may help in identifying potential microRNA annotation errors. Beyond the uses of DeepMirCut, we also provide a new data set of extended precursor sequences for future algorithms to be trained on. While this new dataset is not a replacement for web-accessible microRNA databases such as miRBase (Kozomara et al., 2019), it complements them well, by providing the extended context beyond precursor sequences. We expect future studies to train new architectures on our extended precursor dataset to improve performance of this microRNA cut-site-labeling task. Our extended precursor dataset includes data for plant microRNAs, which were not used in our training or evaluation in this study, and could be studied in future investigations. Future work could incorporate cut site prediction into microRNA discovery pipelines to test if the ends of deep sequencing reads map to strong predicted cut sites.

## Methods

### Data Preprocessing

All microRNA GFF annotations files were downloaded from miRBase v22.1, and then used to locate precursor sequences and the surrounding genomic context for each species. Genome FASTA files were downloaded from various sources including NCBI Assembly and organism-specific genome resources when needed. Supplementary Table S1 lists the download location for each organism. Precursor sequences were extracted from each genome along with a buffer sequence extending 300nt upstream and 300nt downstream. The buffer sequence was shorter in cases where less buffer was available and truncated in cases where it would overlap neighboring microRNA precursors. Cleavage-sites were determined by predicting the secondary structure of the original precursor sequences found on miRBase using RNAfold (Hofacker et al., 1994) and identifying the arm where each mature microRNA was located. Examples where mature microRNA products overlap the portion of the secondary structure prediction corresponding to the hairpin loop were removed to avoid ambiguity in cleavage-sites corresponding to each microRNA. In several cases either the name or location of the miR was inconsistent between the miRBase GFFs and the miRBase FASTA files and in a few cases defunct miRs were present in the miRBase GFFs. In order to improve testability, microRNAs were dropped whenever there was a naming inconsistency between GFF and FASTA files or an inconsistency between the annotation and genomic sequence (70 loci in total). Sequences from *Brassica napus*, *Schistosoma japonicum*, *Schmidtea mediterranea*, and *Triticum aestivum* were excluded from the set because of difficulties in finding versions of the genome that corresponded to locations of each sequence within the miRBase GFF files.

### Performance Metrics

Precision, recall, and F-score all give the same measurement due to DeepMirCut applying a single label for each cut site; therefore, we use perfect match fraction (PMF) and positional shift error (PSE) to evaluate performance. Perfect match fraction is the fraction of predictions for a particular type of cut site that are correctly labeled. Positional shift error is the average absolute value of the distance that a cleavage site prediction is shifted from the annotated position.

$$PMF=\;\frac{Number\;of\;Examples\;with\;Cutsite\;Correctly\;labeled}{Total\;Examples}$$

$$PSE=\;\frac{\sum_{example}\left|Predicted\;Position-Annotated\;Position\right|}{Total\;Examples}$$

### Hyperparameter Tuning

Hyperparameters for three different models were tuned using a training set composed of different input data. The first approach used RNA sequence data only. The second approach used RNA sequence and the dot-bracket sequence corresponding to the predicted secondary structure. The third approach used RNA sequence and the enhanced bpRNA structure array (Danaee et al., 2018). Hyperopt (Bergstra et al., 2013) was used to search for a model producing an optimal perfect match fraction with an embedding dropout between 0 and 0.5, an embedding dimension of 32, 64, 96, 128, or 160 units, a first bidirectional LSTM layer with 64, 128, 192, 256, or 320 units, a second bidirectional LSTM layer with 0, 64, 128, 192, 256, or 320 units, a learning rate for the adam optimizer between 0.00001 and 0.1, and an epsilon between 10^–10^ and 10^–4^. The top 10 models identified by hyperopt were retrained 20 times and a model for each of the three training sets was chosen based on median PMF (see Supplementary Figures S2–S4). After this analysis, we determined that the best model was trained on RNA sequence and enhanced bpRNA structure array.

### Point Mutation Analysis

A point mutation analysis was performed by mutating every nucleotide from −5nt upstream to 5nt downstream of each cut site in the test set. We applied DeepMirCut to predict cut sites on mutated and unmutated datasets and returned the decision values for the annotated cut sites. The mean difference between decision values for mutated nucleotides vs unmutated nucleotides and unmutated vs mutated nucleotides was used to evaluate the effects that mutations at each position would have on the model’s ability to predict cleavage sites.

A second point mutation analysis was performed by mutating each of the characters within the bpRNA structure array from −10 nt upstream to 10nt downstream of each cut site within the test set. The possible characters in the bpRNA structure array are L for left base pair, R for right base pair, H for hairpin loop, B for bulge, I for internal loop, and M for multiloop. DeepMirCut was run on the mutated and unmutated datasets and the mean difference between decision values for each cut site was used to evaluate the effects that mutations at each position of the bpRNA sequence array would have on the model’s ability to predict cleavage sites.

Heatmaps were generated by making every possible point mutation to each of the sequences in the test set, where $s_{i,p}$ corresponds to the nucleotide at position p for sequence i . The value $H_{n,p}$ for the heat map corresponds to a mutation to nucleotide n at position p , and has the value of the mean difference in decision value between characters in the mutated and unmutated sets:

$$H_{n,p}=\frac{\sum_{i}1_{\left[si,p\neq n\right]}\times \left(D_{i,n,p}-D_{i,s_{i,p},p}\right)+1_{\left[s_{i,p}=n\right]}\times \sum_{m\in M_{i,p}}\left(D_{i,n,p}-D_{i,m,p}\right)}{\sum_{i}1_{\left[s_{i,p}\neq n\right]}+\sum_{m\in M_{i,p}}1_{\left[s_{i,p}=n\right]}}$$

where $M_{i,p}$ is the set of valid character mutations for example i at position p , and $D_{i,n,p}$ is the decision value returned for the annotated cut site of example i when the character n is used in position p of the sequence. Note that this average includes mutations away from as well as toward the original character $s_{i,p}$ , which is accounted for by the indicator functions. We identified statistically significant point mutations using a paired difference t-tests on all position/character combinations. We restricted the statistical significance test of the point mutation such that the character had an occurrence of at least 5% at that position in the training set to ensure that the model had seen enough examples to predict the effect of the mutation. We used weblogo (Crooks et al., 2004) to create sequence logos for the unmutated nucleotide and bpRNA sequences spanning each cut site. Sequence logos were used to compare the frequency of occurrence within the unmutated sets to the point mutation analyses.

### Identification of Cleavage Sites for Unannotated Mature microRNAs

In order to test the performance of DeepMirCut on microRNAs with only one annotated mature microRNA, wildtype MCF-7 total cell content (GSE31069) and MCF-7 cell fractions (GSE31069) were downloaded from GEO (Barrett et al., 2010). We identified 904 human microRNAs within our dataset with unique sequences that had only one annotated microRNA and shared less than 80% identity with the training set. Reads from the MCF-7 cell lines were mapped to the Human genome (hg38), and the cleavage sites of the unannotated mature microRNAs were predicted from the location of the read mappings using the miRPreprocess script found in miRWoods (Bell et al., 2019). We filtered out microRNAs with fewer than 5 reads in order to reduce the likelihood that cut sites were identified from spurious reads. The remaining microRNAs were split into two test sets consisting of 9 microRNAs with unannotated products on the 5′ arm and 11 microRNAs with unannotated products on the 3′ arm. We applied ensemble DeepMirCut to each set and evaluated performance against cut sites identified by miRPreprocess.

### Comparison with LBSizeCleav and PHDCleav

The original program only performs 5-fold cross validation on a set of human miRs from miRBase. To perform a direct comparison, we adapted the original code to train and test on our datasets. In doing so, made minimal modifications to avoid changing how the original implementation generated sequence and structure patterns from microRNA precursors, and only removed the 5-fold cross validation so that a input train and test data set could be used.

We had to modify the way DeepMirCut makes predictions in order to compare with other programs. One issue with comparing DeepMirCut to PHDCleave and LBSizeCleav is that they only train and test using positive examples that are at each cut site and negative examples that are exactly 6 nt away from the cut site. To make our comparison fair, we only measured performance at these positions ignoring the remainder of the sequence. A second issue is that DeepMirCut predicts each cut site based on where the decision value reaches its peak. This peak is usually at or near the cut site but will often be lower than a default cutoff score of 0.5. To solve this issue, we normalize by the highest decision value over the length of the precursor. Positive predictions were defined as a decision value greater than 0.5, and negative predictions as less 0.5.

## Data Availability

All microRNA extended precursor sequence data and the DeepMirCut software are available at https://github.com/JimBell/deepMirCut and https://github.com/JimBell/deepMirCut_data.
