# Supplementary material for: Predicting Drosha and Dicer Cleavage Sites with DeepMirCut
Source: Front Mol Biosci. 2022 Jan 24;8:799056. doi: 10.3389/fmolb.2021.799056 (PMC8819831; doi:10.3389/fmolb.2021.799056)
Supplement: Supplementary file 3 [file DataSheet1.PDF]

Supplementary Material for:

Predicting Drosha and Dicer cleavage sites with DeepMirCut

Jimmy Bell[1], David Hendrix[1,2]

1 School of Electrical Engineering and Computer Science, Oregon State University
2 Department of Biochemistry and Biophysics, Oregon State University

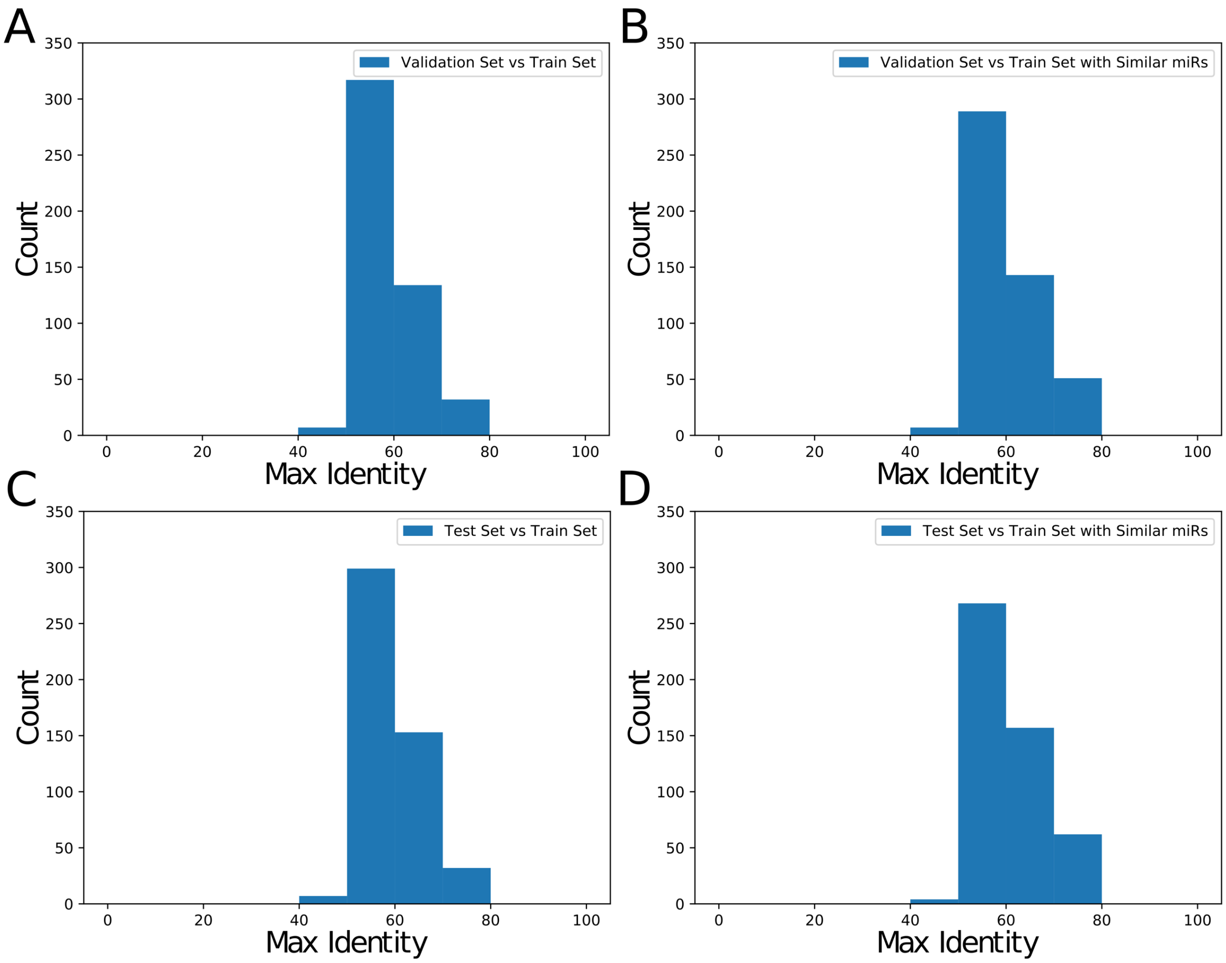

Supplementary Figure 1. A comparison of sequences between data sets to confirm all sequences were below the identity threshold of 0.8. ***A.*** *Validation set compared to training set* ***B.*** *Validation set compared to training set with similar miRs.* ***C.*** *Test set compared to training set.* ***D.*** *Test set compared to training set with similar miRs.*

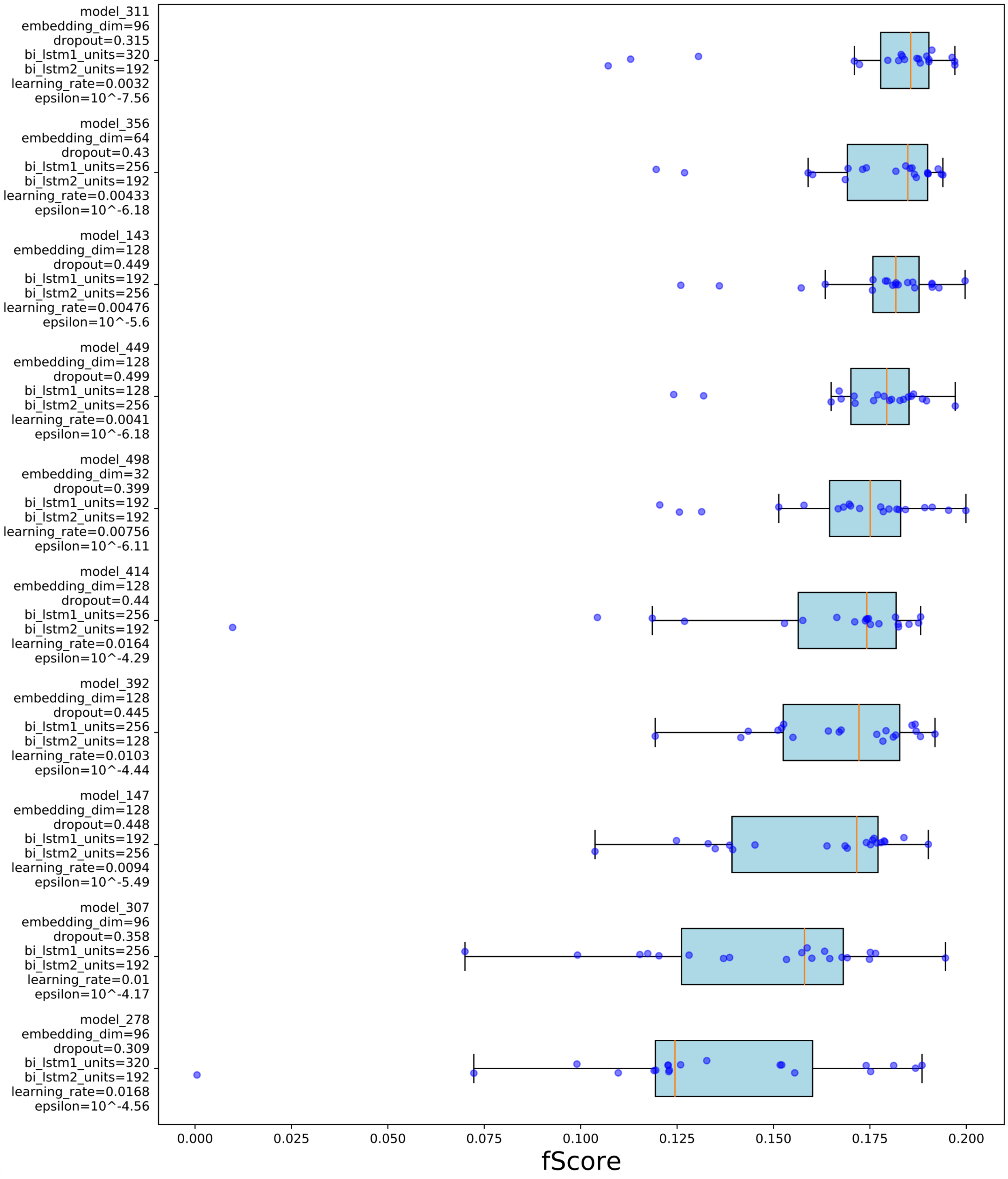

Supplementary Figure 2: Results from hyperparameter tuning, showing the top 10 models trained using sequence only.

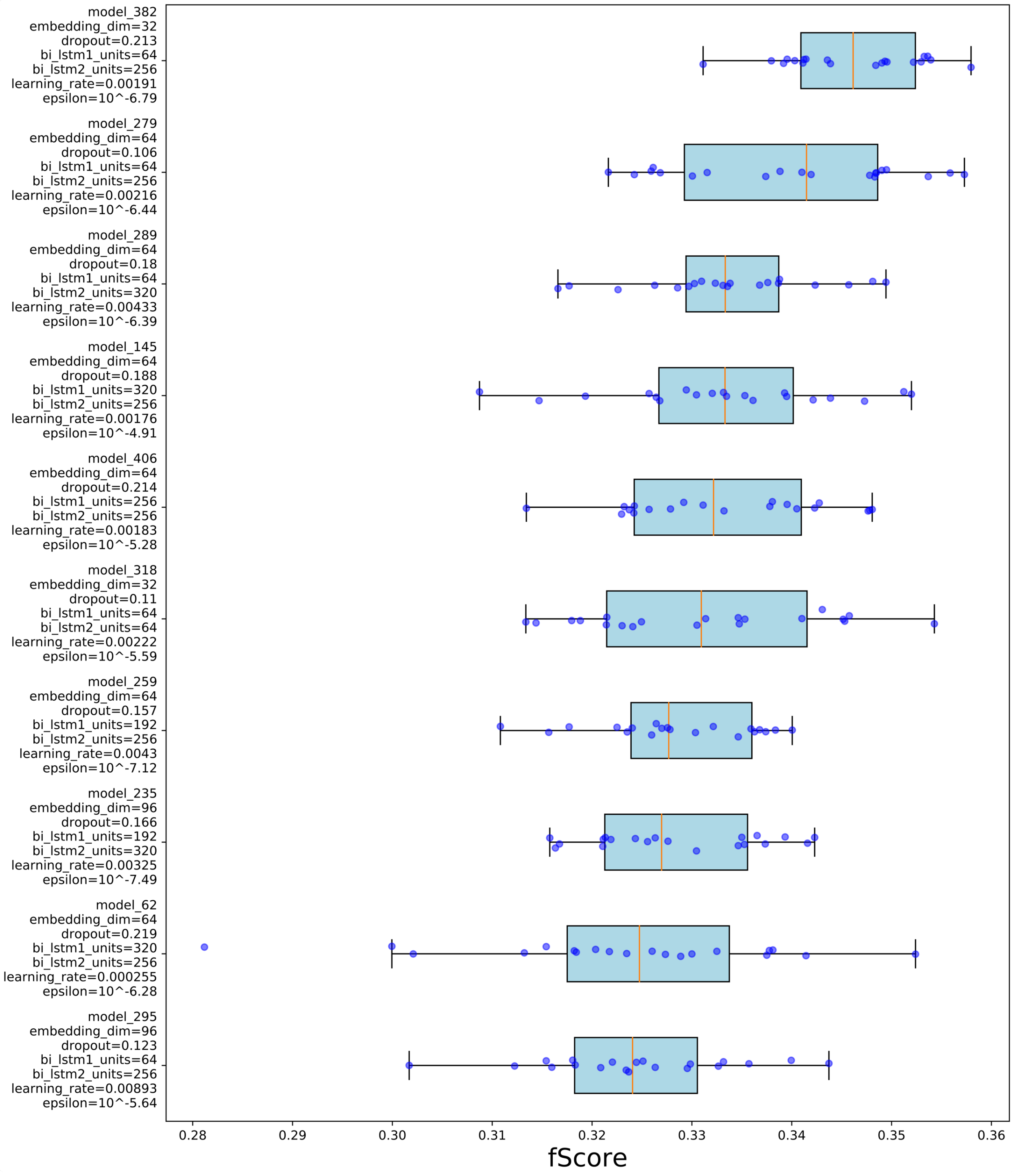

Supplementary Figure 3. Results from hyperparameter tuning, showing the top 10 models trained using sequence and fold.

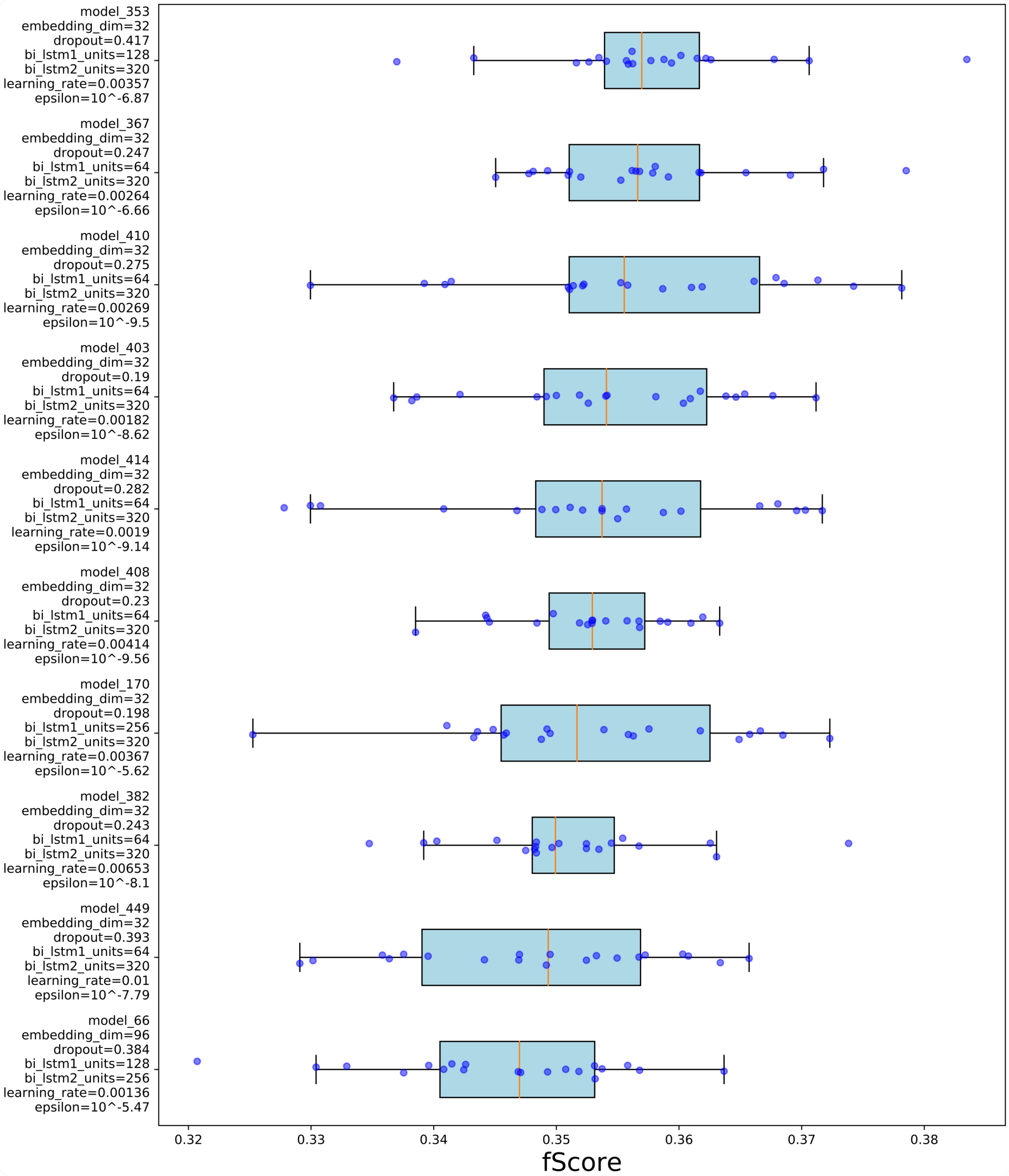

Supplementary Figure 4. Results from hyperparameter tuning, showing the top 10 models trained using sequence and fold context.

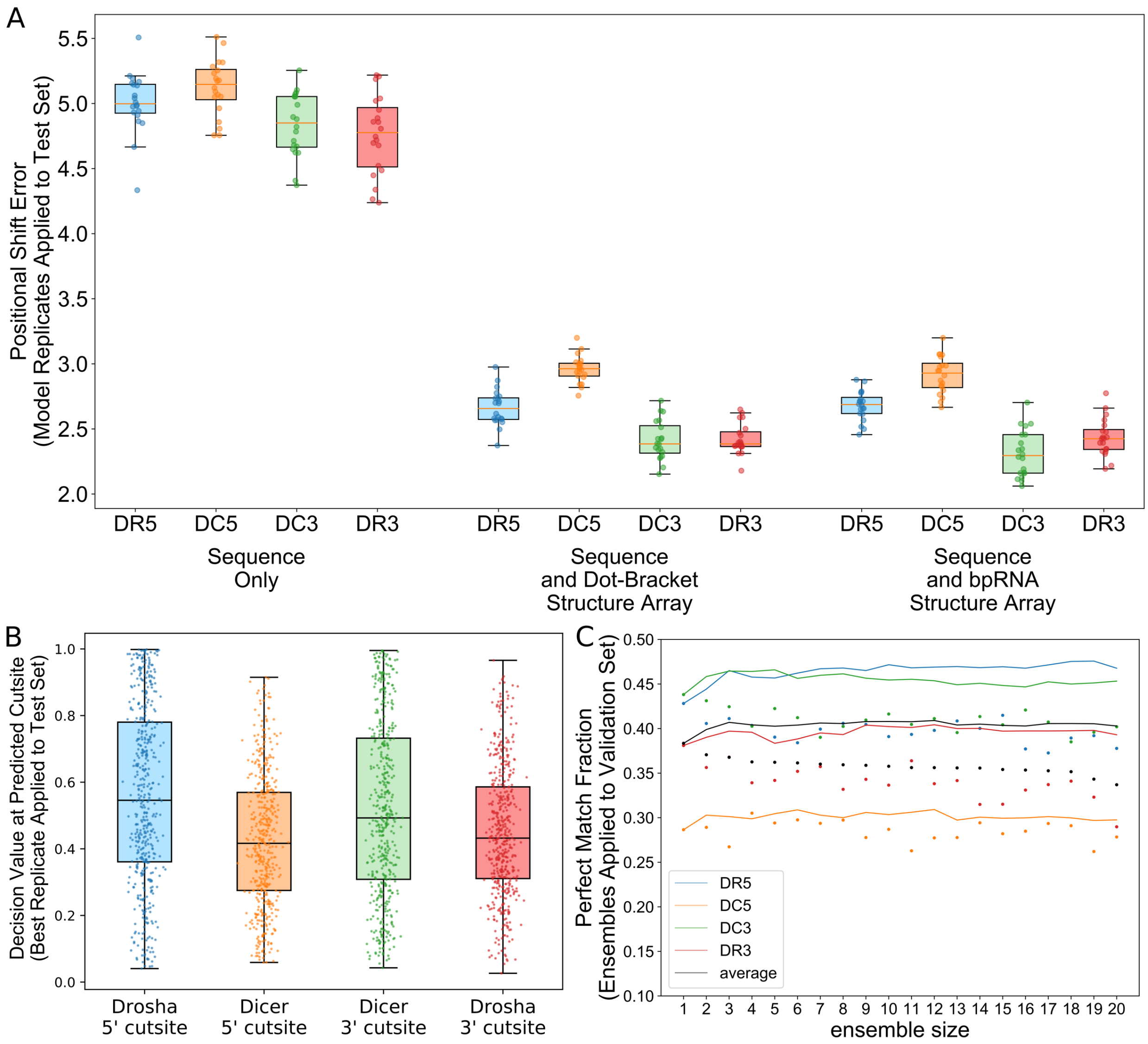

Supplementary Figure 5. Performance of Replicates and the ensemble. ***A.*** *Positional shift error measured for each model replicate.* ***B.*** *Decision value for the best replicate trained on sequence and bpRNA fold context.* ***C.*** *The perfect match fraction for ensembles of increasing sizes when applied to the validation set.*

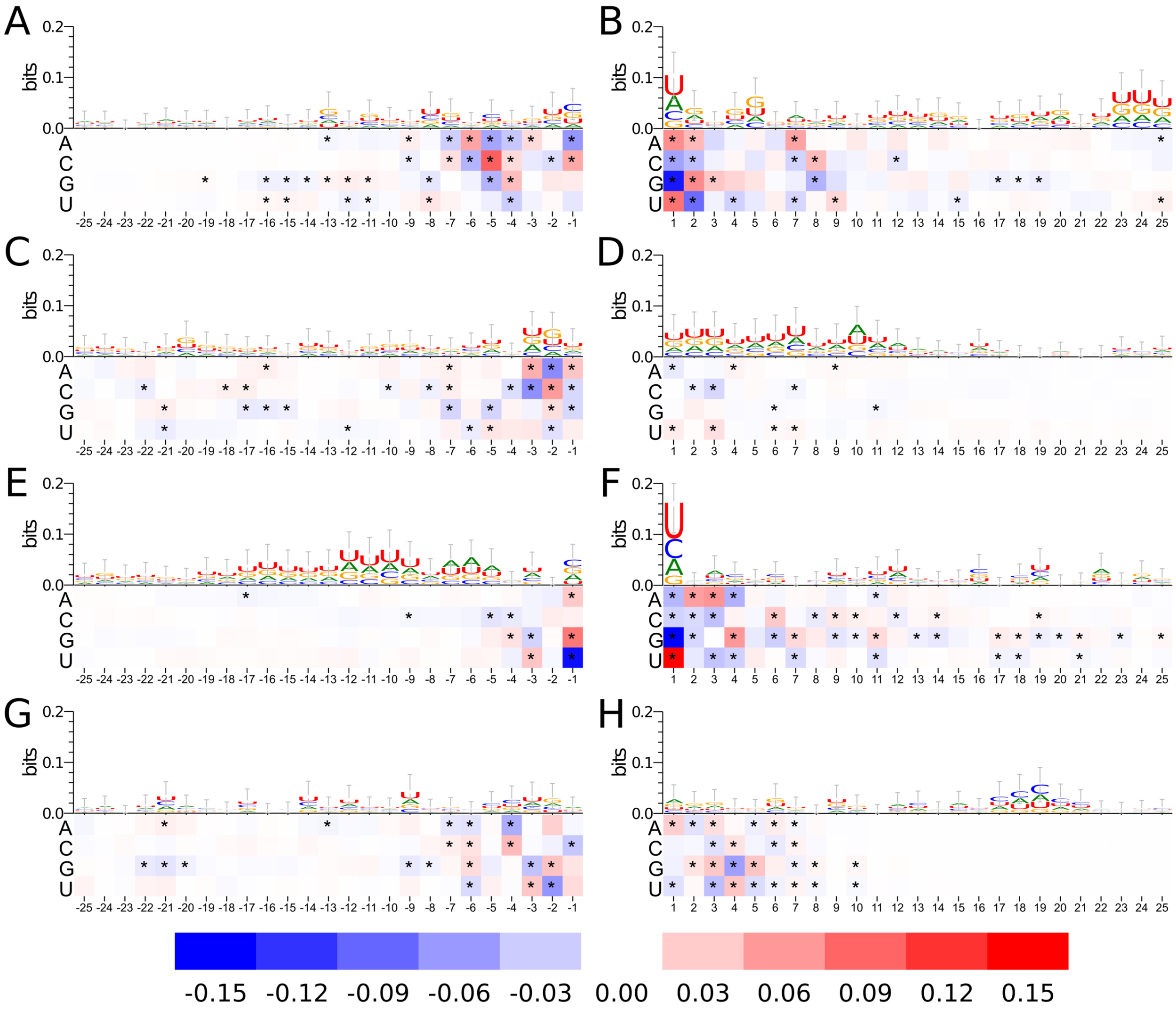

Supplementary Figure 6. Point mutation analysis for nucleotides surrounding cut sites in the test set. Heatmaps showing the average change in decision value due to point mutations for nucleotides ***A.*** *upstream of DR5,* ***B.*** *downstream of DR5,* ***C.*** *upstream of DC5,* ***D.*** *downstream of DC5,* ***E.*** *upstream of DC3,* ***F.*** *Downstream of DC3,* ***G.*** *upstream of DR3,* ***H.*** *and downstream of DR3.*

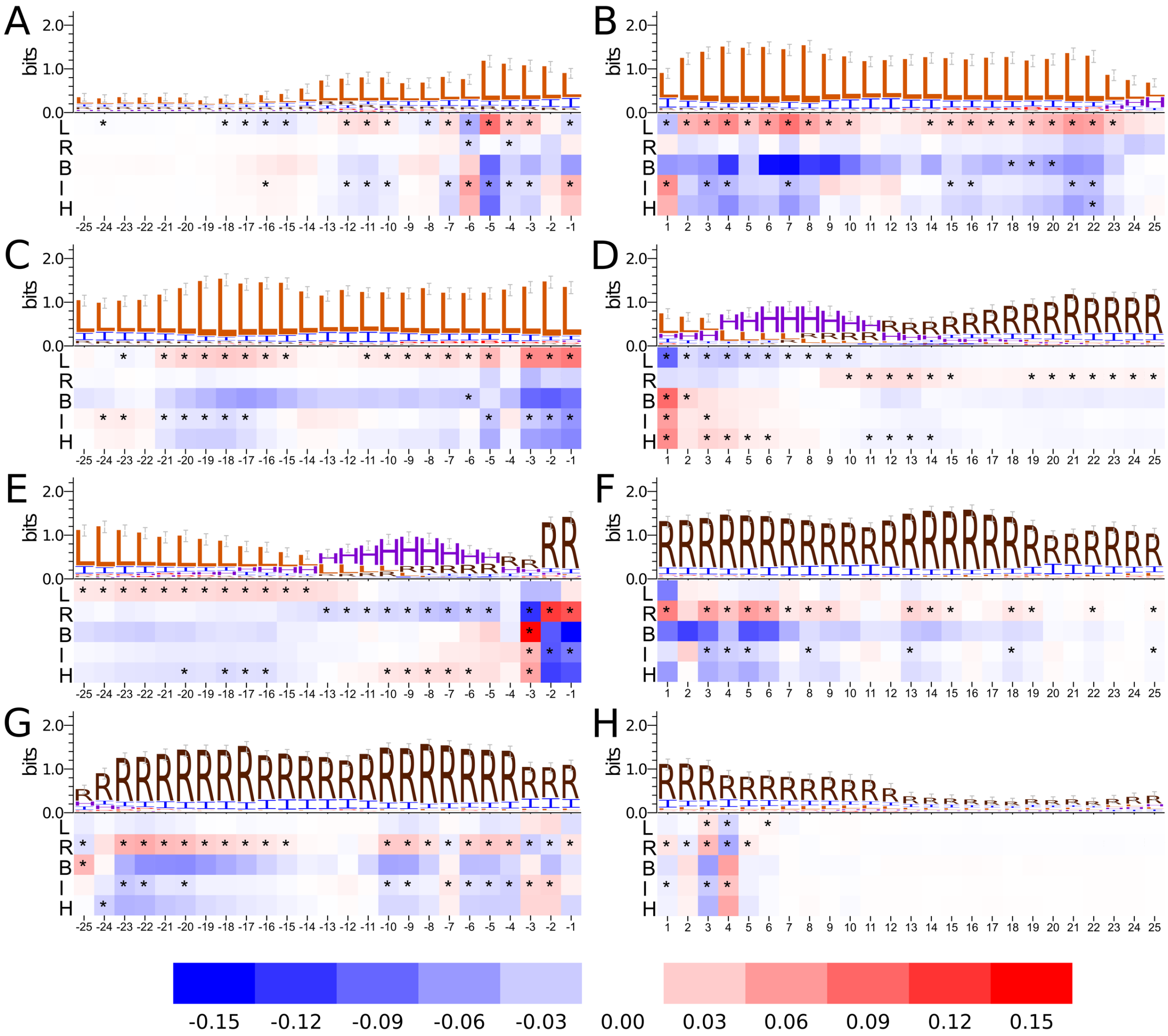

*Supplementary Figure 7. Point mutation analysis for bpRNA sequence. Heatmaps showing the average change in decision value due to point mutations within the enhanced bpRNA sequence for positions **A.** upstream of DR5, **B.** downstream of DR5, **C.** upstream of DC5, **D.** downstream of DC5, **E.** upstream of DC3, **F.** Downstream of DC3, **G.** upstream of DR3, **H.** and downstream of DR3.*

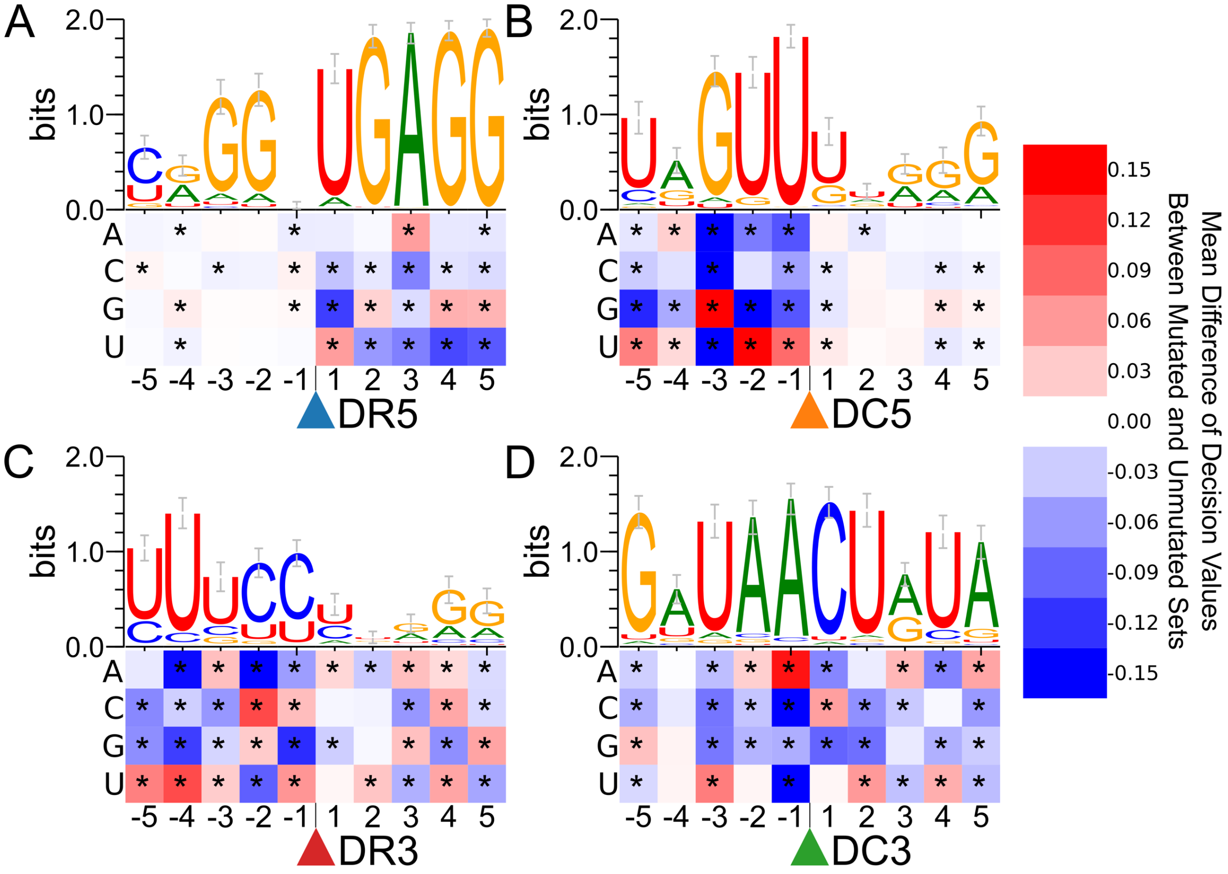

Supplementary Figure 8: Point mutation analysis for nucleotides surrounding cut sites in members of the let-7 family. Heatmaps show the average change in decision value due to point mutations for nucleotides surrounding cleavage sites of ***A.*** *Drosha on the 5' arm,* ***B.*** *Dicer on the 5' arm,* ***C.*** *Drosha on the 3' arm, and* ***D.*** *Dicer on the 3' arm.*

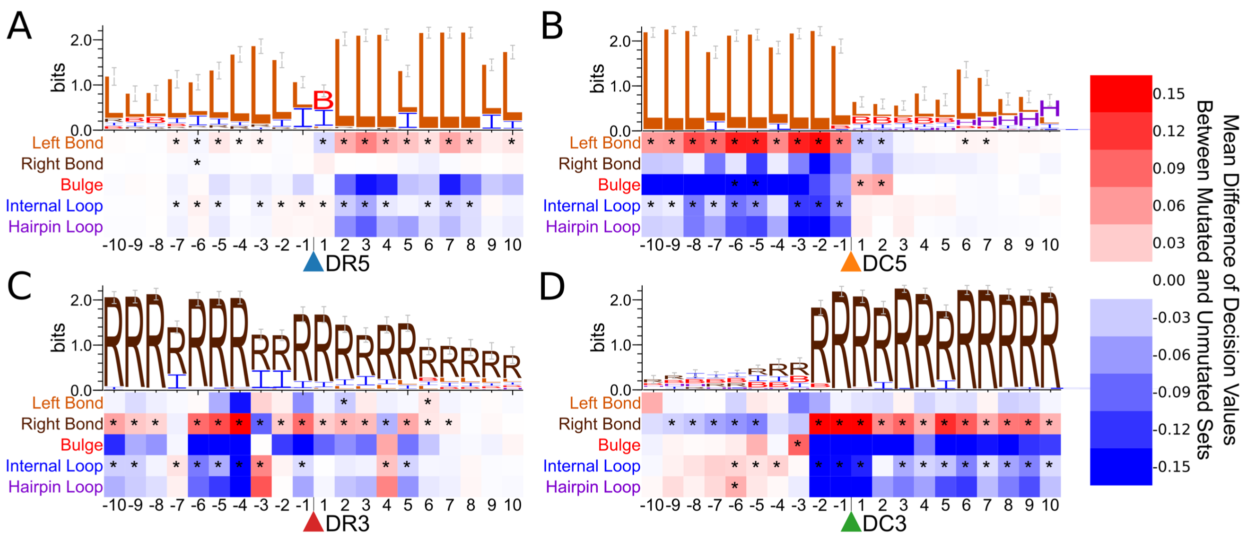

Supplementary Figure 9: Point mutation analysis for bpRNA sequence for cut sites in the let-7 family. Heatmaps show the average change in decision value due to point mutations within the enhanced bpRNA sequence surrounding cleavage sites of ***A.*** *Drosha on the 5' arm,* ***B.*** *Dicer on the 5' arm,* ***C.*** *Drosha on the 3' arm, and* ***D.*** *Dicer on the 3' arm.*

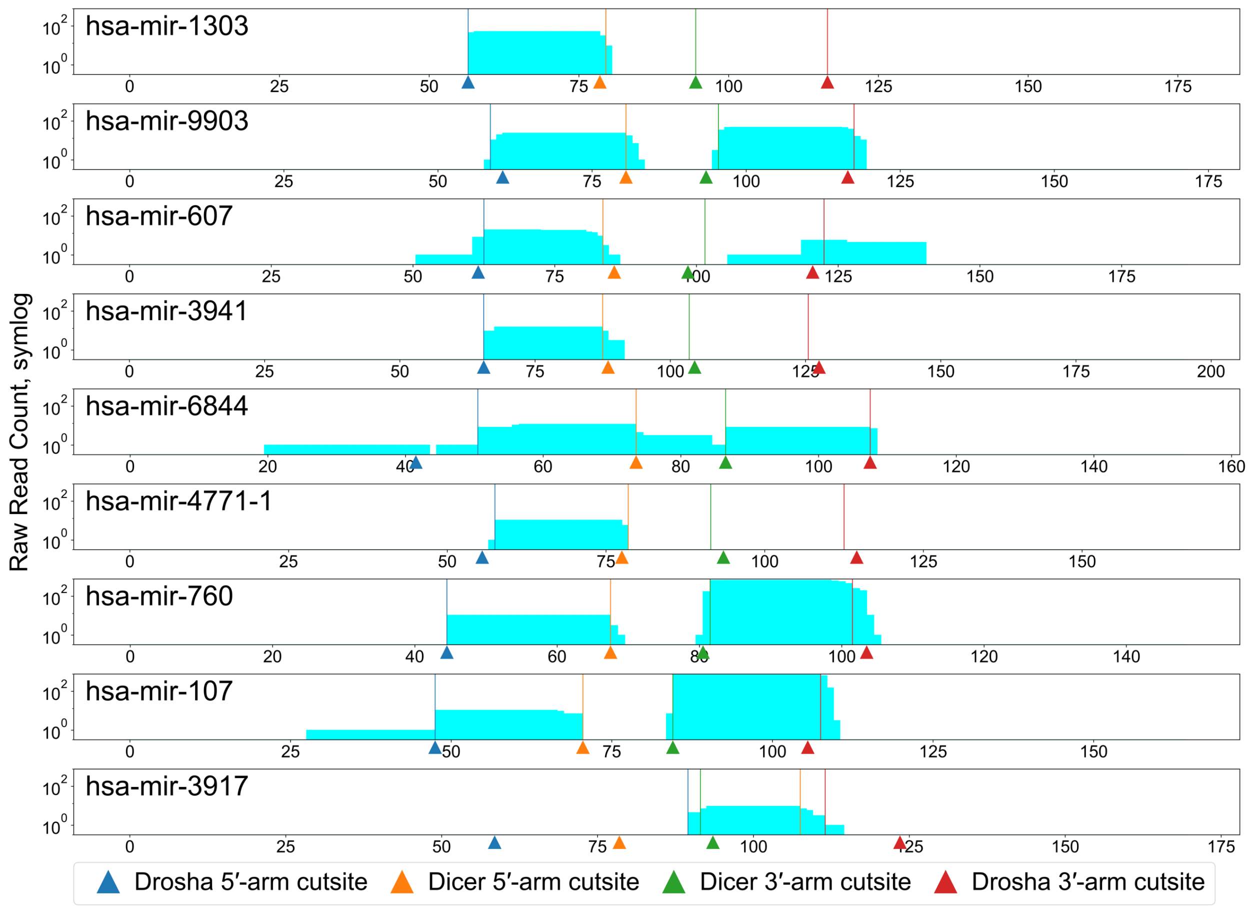

Supplementary Figure 10. Mapped read counts and cut site predictions for known microRNA precursors with unannotated microRNAs on the 5' arm. Vertical lines show the position of cut sites that are either annotated in miRBase or predicted using the miRPreprocess.pl script from miRWoods. Arrows show the location of cut sites predicted by ensemble DeepMirCut.
